# Supplementary figures and images for: Concurrent infection with Mycobacterium tuberculosis confers robust protection against secondary infection in macaques
Source: PLoS Pathog. 2018 Oct 12;14(10):e1007305. doi: 10.1371/journal.ppat.1007305 (PMC6200282; doi:10.1371/journal.ppat.1007305)

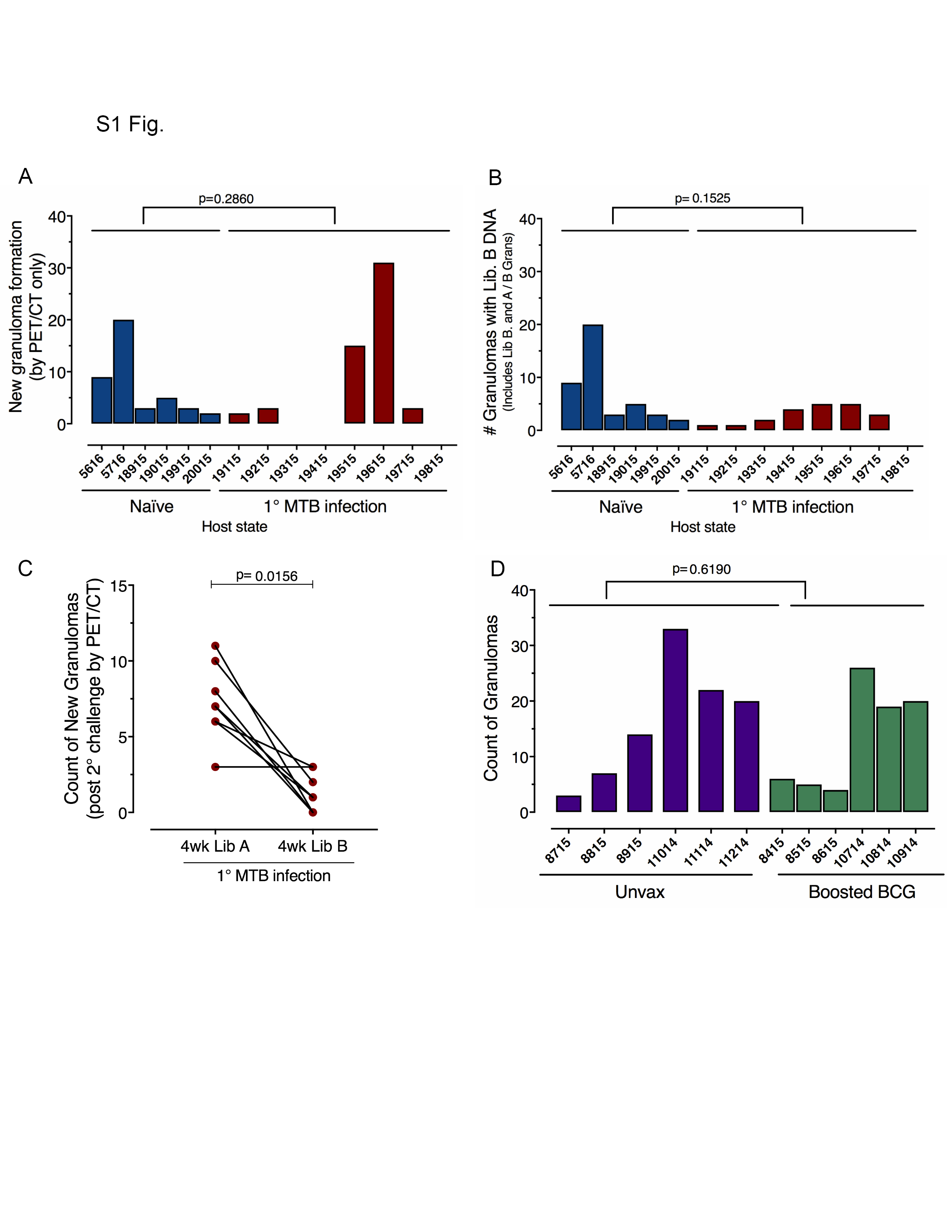

Supplement: S1 Fig — (a) All new granulomas seen by PET CT in naïve and reinfected macaques at 4 weeks post-Library B infection. (b) All granulomas with Library B DNA in naïve or reinfected macaques. This count includes both newly established and pre-existing granulomas that contained library B in the reinfection cohort. Monkey ID: 19815 had no detectable library B in any tissue. (c) Number of new granulomas seen by PET CT established by library A or library B 4 weeks post-infection in the same animal. p = 0.0156, Wilcoxon-matched pairs signed rank test. (d) Number of granulomas seen by PET CT at 4–5 weeks post-Mtb Erdman infection between unvaccinated and BCG+H56 vaccinated macaques. Statistics: Mann-Whitney test. (TIF) [file ppat.1007305.s001.tif]

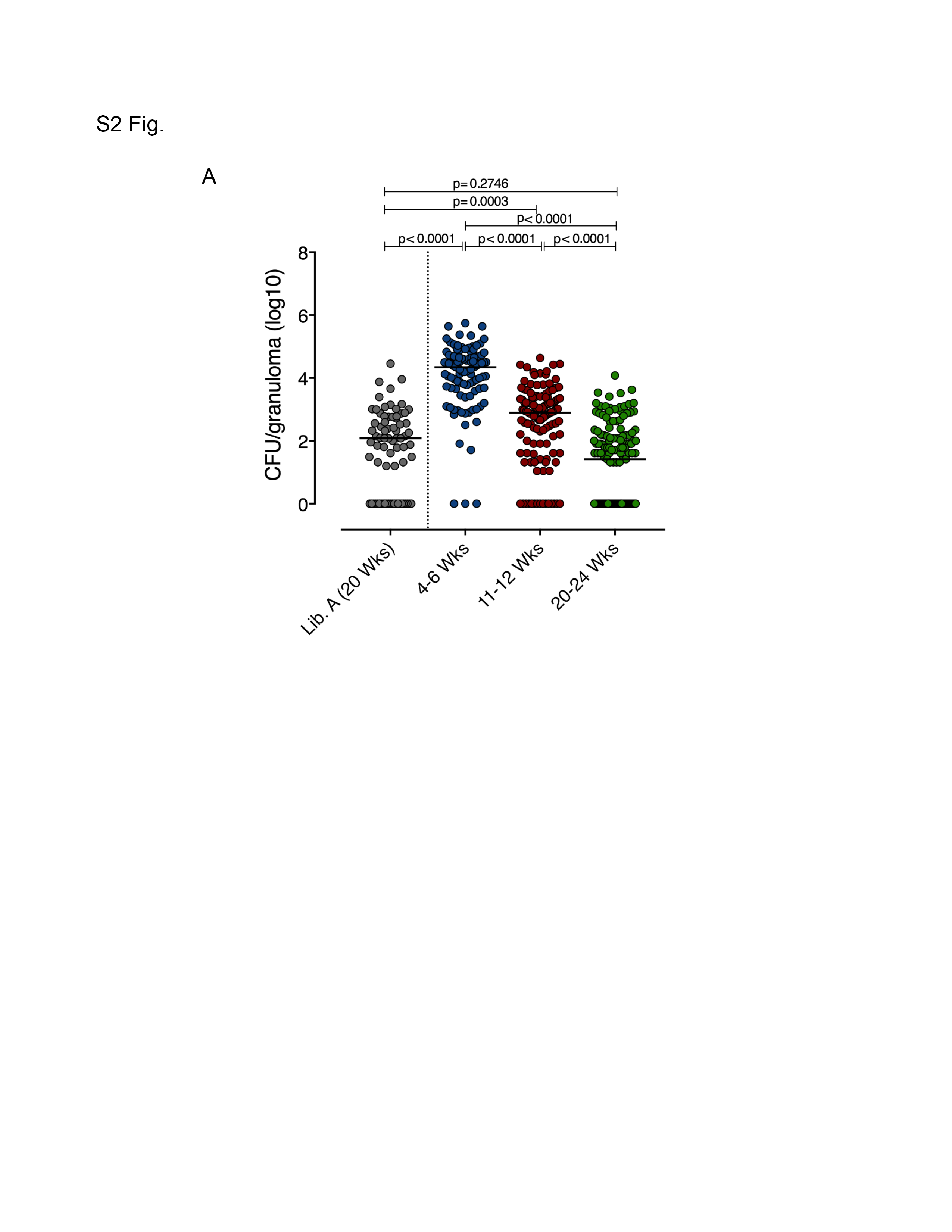

Supplement: S2 Fig — CFU/granuloma was compared from Mtb Erdman infected macaques at 4–6, 11–12, and 20–24 weeks post-infection, using historical controls (N = 17). This is similar to data published in Lin, Ford, et al, but using a different set of monkeys. On the left side of the graph, CFU/granuloma of library A in our reinfected animals is shown (N = 8); these values are similar to the values of Erdman at 20–24 weeks. (TIF) [file ppat.1007305.s002.tif]

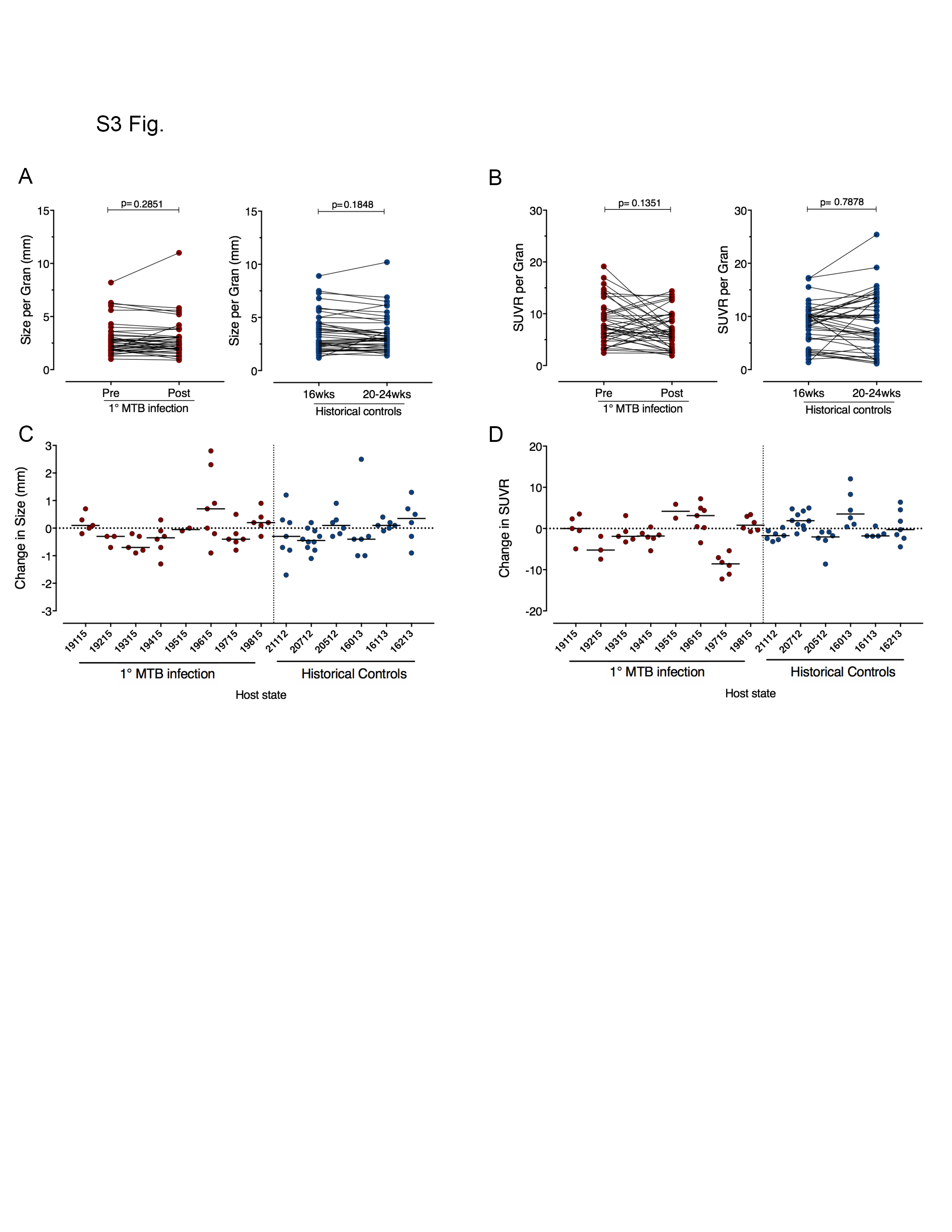

Supplement: S3 Fig — (A) Granulomas do not change significantly in size after infection with Library B in the reinfection animals (p = 0.2851). Similarly, granulomas from animals infected with the Erdman strain do not change significantly in size from 16 to 20 (or 24) weeks post-infection (p = 0.1848). (B) Previously established (library A) granulomas do not significantly increase or decrease in FDG activity (SUVR) after reinfection with Library B (p = 0.1351). Likewise, in Erdman-infected historical controls, granulomas do not significantly change in SUVR (p = 0.7878) from 16 to 20 weeks post-infection. (C) Change in size (mm) per granuloma by animal. (D) Change in FDG activity (SUVR) per granuloma by animal. Each symbol represents a granuloma. Statistics: Wilcoxon matched-pairs signed rank test. (TIF) [file ppat.1007305.s003.tif]

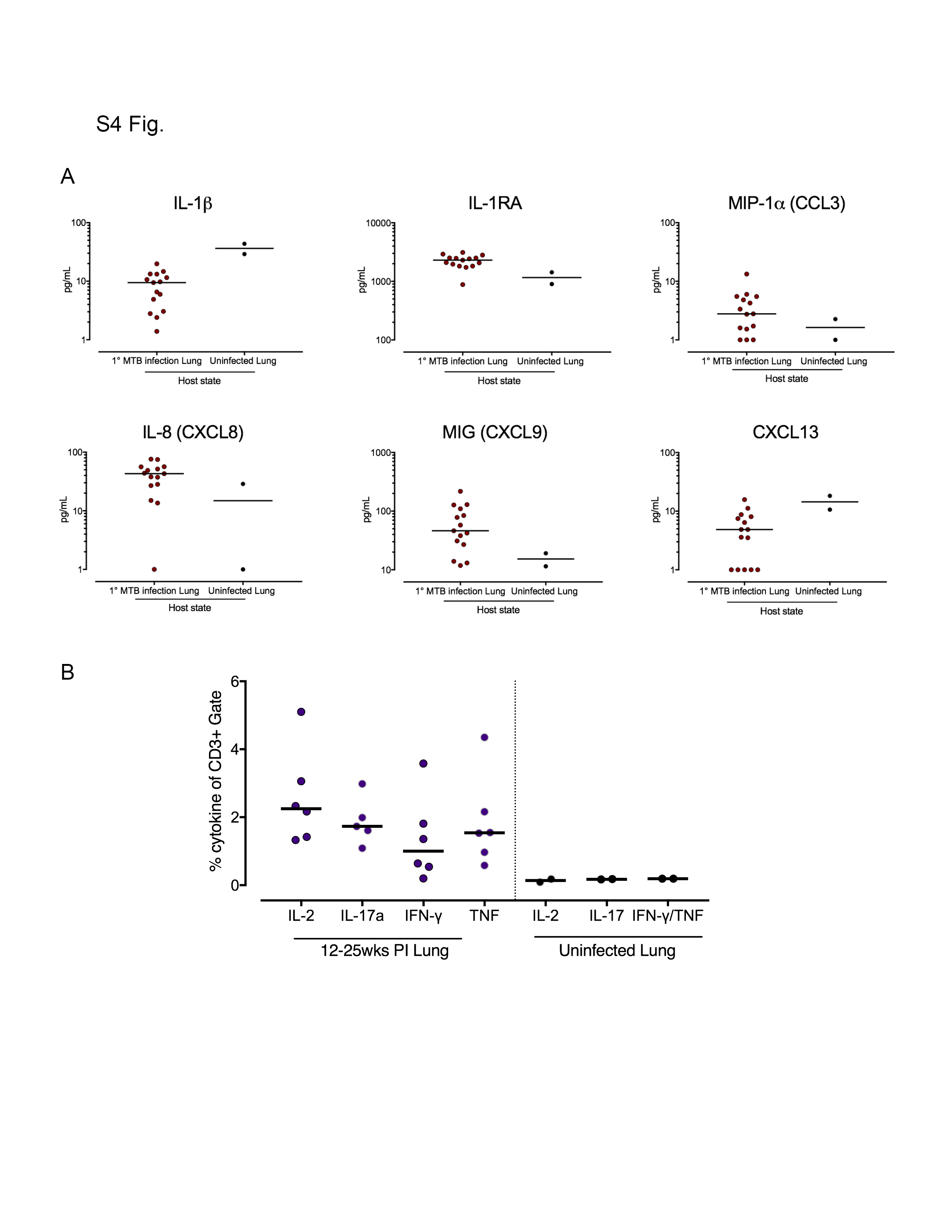

Supplement: S4 Fig — A. Luminex analysis on supernatant from uninvolved (no granuloma) lung tissue was compared between reinfected macaques and an uninfected macaque. B. Using a separate set of macaque lung tissue (20–24 weeks post-infection but not reinfected), the T cell responses following ESAT-6 and CFP10 stimulation were assessed by flow cytometry, and compared to an uninfected macaque. No statistics were performed, due to the small sample size for the uninfected macaque lung. (TIF) [file ppat.1007305.s004.tif]

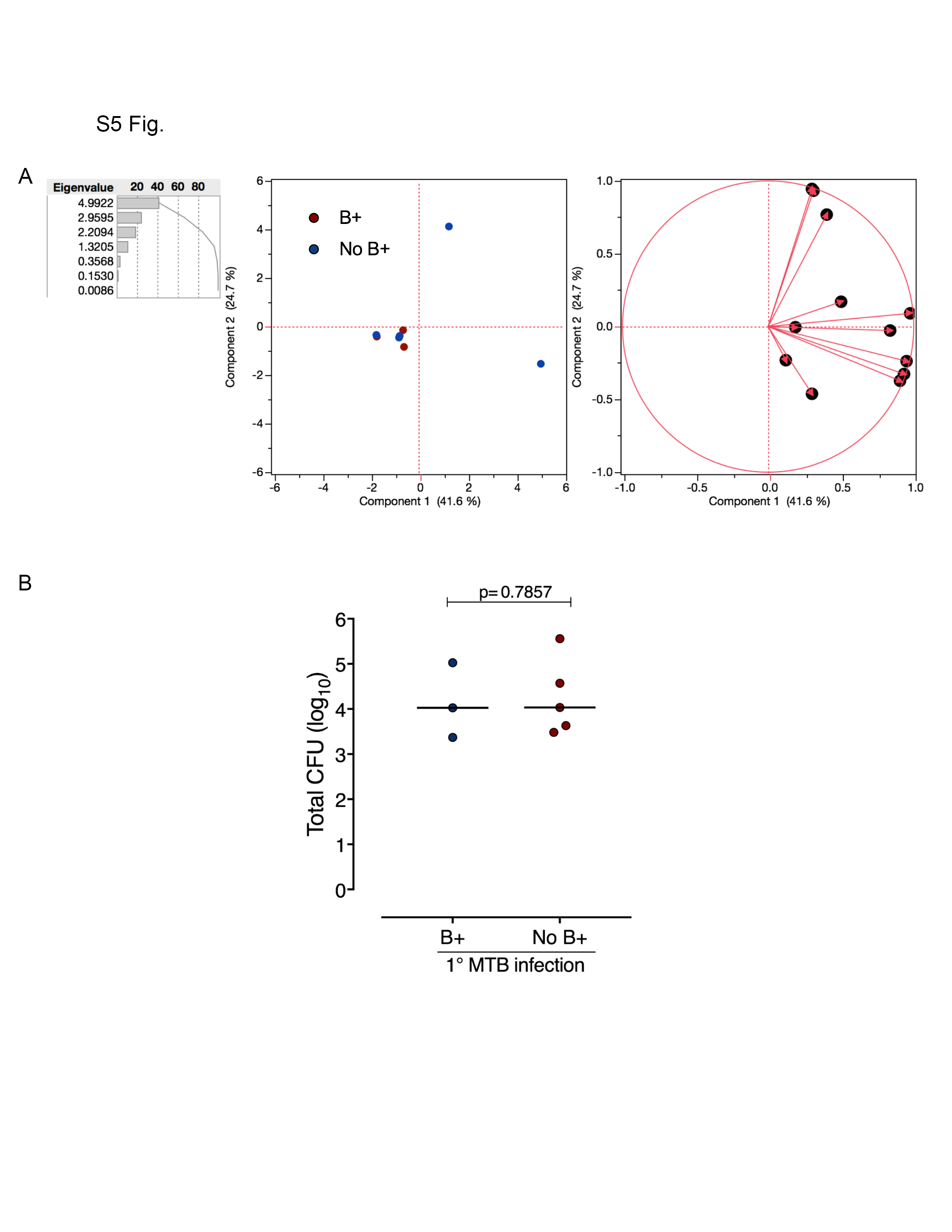

Supplement: S5 Fig — (A) Principal Components Analysis of PBMC data. Far left is the Eigenvalue Pareto Plot showing the cumulative percentage of variation accounted for in each principal component. Middle is a scatterplot of the first two components color-coded for animals with CFU-positive B granulomas (blue), and those without (red). There is no obvious clustering of these groups. Far right is a loading plot showing the correlations of the original variables to the first two principal components. B. Total thoracic CFU (log10) is similar between animals with and without CFU-positive Library B granulomas (p = 0.7857, Mann-Whitney test). (TIF) [file ppat.1007305.s005.tif]
