# Supplementary material for: Concurrent infection with Mycobacterium tuberculosis confers robust protection against secondary infection in macaques
Source: PLoS Pathog. 2018 Oct 12;14(10):e1007305. doi: 10.1371/journal.ppat.1007305 (PMC6200282; doi:10.1371/journal.ppat.1007305)
Supplement: S1 Table — (PDF) [file ppat.1007305.s006.pdf]

S1 Table. Parameters of macaque infection, serial imaging, bacterial burden and disease pathology.

Animal ID	Strain	Infection Dose (CFU[a])	Time to Nx (wks)	Gross Pathology Score	Total CFU	PET/CT Scans (wks)
18915	Library B 2.0 Barcoded Mtb Erdman	6	4	7	318,039	2, 3
19015		6	4	17	1,738,747	2, 4
19915		5	4	19	564,310	2, 4
20015		5	5	11	104,809	3, 5
5616		10	5	32	370,409	4, 5
5716		10	4	20	461,740	3, 4
19115	1°: Library A 2.0 Barcoded Mtb Erdman<br>2°: Library B 2.0 Barcoded Mtb Erdman	1° = 5<br>2° = 5	20	23	37,756	4, 6, 8, 12, 15, 18, 20
19215		1° = 5<br>2° = 5	20	22	6719	4, 6, 8, 12, 15, 18, 20
19315		1° = 5<br>2° = 5	21	12	12,196	4, 6, 8, 12, 15, 18, 21
19415		1° = 5<br>2° = 5	21	22	4,334	4, 7, 8, 13, 15, 18, 21
19515		1° = 8<br>2° = 10	20	38	361,420	4, 8, 12, 16, 20
19615		1° = 8<br>2° = 10	20	57	122,872	4, 8, 12, 16, 20
19715		1° = 8<br>2° = 10	21	34	10,600	4, 6, 8, 12, 16, 19, 21
19815		1° = 8<br>2° = 10	21	28	3,331	5, 6, 8, 13, 16, 19, 21

[a]CFU=Colony forming units

S2 Table. Number of granulomas recovered with DNA identifiers for Library A or B.

Library Tag	Reinfection	Naïve
# of A Grans	74	0
# of B Grans	12[1]	26
# of A/B Grans	9[1]	0

[1]One granuloma had library B detected in homogenate DNA but grew up only library A by CFU from scrapate.

S3 Table. Percentage of Lib. B of total lung bacterial burden.

Animal ID	Host State	Lib. B Gran / Total Lung CFU (%)
	Naïve	
5615		100.00
5716		100.00
18915		100.00
19015		100.00
19915		100.00
20015		100.00
	1° MTB infection	
19115		0.00
19215		0.00
19315		0.00
19415		6.38
19515		0.00
19615		0.64
19715		0.84
19815		0.00

S4 Table. Parameters of macaque infection, serial imaging, bacterial burden and disease pathology for BCG+H56 Study.

Animal ID	Strain	Infection Dose (CFU[a])	Time to Nx (wks)	Gross Pathology Score	Total CFU	PET/CT Scans (wks)
8415	Mtb Erdman	2	4	15	546,065	2, 3, 4
8515		2	5	14	183,440	2, 3, 4, 5
8615		2	6	9	13,960	2, 3, 4, 6
8715		2	4	25	1,411,990	2, 3, 4
8815		2	5	19	647,820	2, 3, 4, 5
8915		2	5	23	624,465	2, 3, 5
10714		31	4	20	649,695	3, 4
10814		31	5	14	124,560	3, 4, 5
10914		31	5	14	1,192,200	3, 4, 5
11014		31	5	24	2,632,065	3, 4, 5
11114		31	4	21	885,210	3, 4
11214		31	6	13	373,005	3, 4, 6

[a]CFU=Colony forming units
