# Supplementary material for: Concurrent infection with Mycobacterium tuberculosis confers robust protection against secondary infection in macaques
Source: PLoS Pathog. 2018 Oct 12;14(10):e1007305. doi: 10.1371/journal.ppat.1007305 (PMC6200282; doi:10.1371/journal.ppat.1007305)
Supplement: S2 Table — (PDF) [file ppat.1007305.s007.pdf]

S2 Table. Number of granulomas recovered with DNA identifiers for Library A or B.

Library Tag	Reinfection	Naïve
# of A Grans	74	0
# of B Grans	12[1]	26
# of A/B Grans	9[1]	0

[1]One granuloma had library B detected in homogenate DNA but grew up only library A by CFU from scrapate.
