# Supplementary material for: Concurrent infection with Mycobacterium tuberculosis confers robust protection against secondary infection in macaques
Source: PLoS Pathog. 2018 Oct 12;14(10):e1007305. doi: 10.1371/journal.ppat.1007305 (PMC6200282; doi:10.1371/journal.ppat.1007305)
Supplement: S3 Table — (PDF) [file ppat.1007305.s008.pdf]

S3 Table. Percentage of Lib. B of total lung bacterial burden.

Animal ID	Host State	Lib. B Gran / Total Lung CFU (%)
	Naïve	
5615		100.00
5716		100.00
18915		100.00
19015		100.00
19915		100.00
20015		100.00
	1° MTB infection	
19115		0.00
19215		0.00
19315		0.00
19415		6.38
19515		0.00
19615		0.64
19715		0.84
19815		0.00
