# Supplementary material for: Concurrent infection with Mycobacterium tuberculosis confers robust protection against secondary infection in macaques
Source: PLoS Pathog. 2018 Oct 12;14(10):e1007305. doi: 10.1371/journal.ppat.1007305 (PMC6200282; doi:10.1371/journal.ppat.1007305)
Supplement: S4 Table — (PDF) [file ppat.1007305.s009.pdf]

S4 Table. Parameters of macaque infection, serial imaging, bacterial burden and disease pathology for BCG+H56 Study.

Animal ID	Strain	Infection Dose (CFU[a])	Time to Nx (wks)	Gross Pathology Score	Total CFU	PET/CT Scans (wks)
8415	Mtb Erdman	2	4	15	546,065	2, 3, 4
8515		2	5	14	183,440	2, 3, 4, 5
8615		2	6	9	13,960	2, 3, 4, 6
8715		2	4	25	1,411,990	2, 3, 4
8815		2	5	19	647,820	2, 3, 4, 5
8915		2	5	23	624,465	2, 3, 5
10714		31	4	20	649,695	3, 4
10814		31	5	14	124,560	3, 4, 5
10914		31	5	14	1,192,200	3, 4, 5
11014		31	5	24	2,632,065	3, 4, 5
11114		31	4	21	885,210	3, 4
11214		31	6	13	373,005	3, 4, 6

[a]CFU=Colony forming units
